# Comment on Depoorter, L.; Vandenplas, Y. Probiotics in Pediatrics. A Review and Practical Guide. *Nutrients* 2021, *13*, 2176

**DOI:** 10.3390/nu14040724

**Published:** 2022-02-09

**Authors:** Rudolf von Bünau, Andreas Erhardt, Eduard Stange

**Affiliations:** 1 Pharma-Zentrale GmbH, Department of Biological Research, Loerfeldstraße 20, 58313 Herdecke, Germany; 2 Petrus-Krankenhaus, Clinic for Internal Medicine II, Carnaper Str. 48, 42283 Wuppertal, Germany; andreas.erhardt@cellitinnen.de; 3 Happoldstr. 71a, 70469 Stuttgart, Germany; eduard.stange@icloud.com

We read the review by Depoorter et al. [1] recently published in *Nutrients*. With reference to the above-mentioned publication, we would like to point out that the following statement with respect to *E. coli* Nissle is misleading and incorrect: 

“In adults, several studies showed a beneficial effect of *E. coli* Nissle 1917 compared to standard treatment with mesalazine alone in maintaining remission of the disease. Again, these results were not confirmed by any randomize controlled trial (RCT).”

The metaanalysis by Scaldaferri et al. [2] reviewed all four randomized clinical trials with *Escherichia coli* Nissle 1917 (EcN) on maintenance of remission of Ulcerative Colitis in adults and demonstrated equivalence with standard mesalamine (risk ratio 1.08, 95% CI 0.89–1.29). Another metaanalysis by Losurdo et al. [3] confirms the results and conclusions of Scaldaferri et al. with an odds ratio of 1.07, 0.70–1.64. 

Depoorter and Vandenplas claim that these results [3] were not confirmed by any RCT; however, all studies in the metaanalysis actually were RCTs and, furthermore, the reference of Petersen et al. [4] only refers to a trial on another indication, the induction of remission with ciprofloxacin and EcN. 

Considering the multiple and sometimes critical side effects of mesalamine including renal impairment, pancreatitis, pleuritis, pericarditis and myocarditis, *E. coli* Nissle should be classified as an evidence-based and safe alternative in adults with four controlled trials and two metaanalyses supporting this statement. We agree that in children the evidence is inconclusive [5].
